# LC/ESI-MS/MS phytochemical profiling and apoptotic effect of *Haloxylon scoparium* leaf extract on hepatocellular carcinoma

**DOI:** 10.1038/s41598-025-21041-2

**Published:** 2025-10-23

**Authors:** Mosad A. Ghareeb, Boulanouar Bakchiche, Yacine Aouiffat, Tarek Aboushousha, Mohamed Marzouk, Hend Okasha

**Affiliations:** 1 Medicinal Chemistry Department, Theodor Bilharz Research Institute, Kornaish El-Nile, Warrak El-Hadar, Imbaba, P.O. 30, Giza, 12411 Egypt; 2 Laboratory of Biological and Agricultural Sciences (LBAS), Amar Telidji University, Laghouat, 03000 Algeria; 3 Research Unit of Medicinal Plant (RUMP) Attached to Center of Biotechnology (CRBt, 3000, Constantine), Laghouat 03000, Algeria; 4 Department of Pathology, Theodor Bilharz Research Institute, Kornaish El Nile, Warrak El-Hadar, Imbaba, P.O. Box 30, Giza, 12411 Egypt; 5 Chemistry of Tanning Materials and Leather Technology Department, Chemical Industries Research Institute, National Research Centre, 33 El-Bohouth St. (Former El-Tahrir St.), Dokki, Cairo, 12622 Egypt; 6 Department of Biochemistry and Molecular Biology, Theodor Bilharz Research Institute, Kornaish El Nile, Warrak El-Hadar, Imbaba, P.O. Box 30, Giza, 12411 Egypt

**Keywords:** *Haloxylon scoparium*, Hepatocellular carcinoma, LC/ESI-MS/MS, Polyphenols, TNF-α, *In silico* study, Good health and well-being, Biological techniques, Drug discovery

## Abstract

*Haloxylon scoparium*, a plant native to Moroccan Sahara, was investigated for its potential anticancer activity against hepatocellular carcinoma (HCC). The study aimed to evaluate the effects of its methanolic extract on HCC and to conduct detailed chemical analysis using LC-ESI-MS/MS. *In vitro* cytotoxicity was assessed using HepG2 liver cancer cell line. *In vivo* experiments involved inducing HCC in mice with diethylnitrosamine (DEN). The study monitored inflammatory (TNF-α), apoptotic (BAX, Caspase-3, Caspase-8), and oncogenic markers (AFP, Bcl-2) through blood and liver tissue analysis. Liver histopathology was also performed to evaluate tissue-level changes. Mice survival rates were 83.33% in the DEN group and 91.67% in the DEN/*H. scoparium* group. Liver function markers (TBILR, ALP, AST) significantly decreased in the treatment group. TNF-α levels, elevated in DEN-only mice, were notably reduced after treatment. Oncogenic markers showed significant elevation in the DEN group but were decreased in the treatment group, whereas apoptotic markers were significantly elevated after treatment. Histopathology revealed more preserved liver architecture and scattered apoptotic foci in treated mice. Phytochemical profiling identified 27 compounds, including organic acids, phenolic acid derivatives, and flavonoids by LC/ESI-MS/MS. Molecular docking using AutoDock MGLTools 1.5.7 showed strong binding affinities of quercetin and isorhamnetin glycosides with cancer-related proteins (BCL-2, BAX, Caspases, AFP, TNF-α), supporting experimental results. 3D interaction models and box plots confirmed the stability and specificity of ligand–protein interactions. The study concludes that *H. scoparium* extract demonstrates promising multi-target anticancer potential and may serve as a valuable candidate for pharmaceutical development.

## Introduction

*Haloxylon scoparium* (Pomel) Bge. [Synonyms: *H. articulatum* ssp. *scoparium* (Pomel) Batt., *Arthrophytum scoparium* (Pomel) Iljin ex Maire & Weiller, *Hammada articulata* ssp. *scoparia* (Pomel) O.Bolòs & Vigo, and *Salsola articulata* Cav.] belongs to the family Chenopodiaceae of 120 genera and more than 1700 species^1^.

Globally, they are distributed particularly in the salty desert and semi-desert areas in the form of herbs, subshrubs, and shrubs up to rarely small trees. The genus *Haloxylon* Bunge (Incl *Hammada*) includes about 25 species. It is distributed from the Western Mediterranean region to Arabia, Iran, Mongolia, Burma, and Southwest China^2,3^. *H. scoparium* is a small, highly branched halophytic shrub distributed in sandy waste places of Northern Africa (Algeria, Libya, Morocco, Tunisia, Western Sahara, Egypt-Sinai; West tropical Africa, Mauritania) and Asia-Temperate: (Western Asia: Lebanon-Syria, Palestine)^4^. In traditional medicine, *H. salicornicum* is reported for diabetes^5^, as an antiseptic and anti-inflammatory^6^. In Oman, its stems are used as a mordant for dyeing wool in traditional weaving. In addition, *H. scoparium* is used to treat eye disorders^1^. In Morocco, infusion and powder infusion of the aerial parts of *H. scoparium* are used for their antidiabetic effects^7^. This popular plant is widely used as a decoction, infusion, or cataplasm to treat hypertension, cutaneous neoplasms, dermatitis, diabetes, food poisoning, rheumatoid arthritis, osteoarthritis, scabies, injury healing, indigestion, stomachache, gastroenteritis, and cold^8^. Moreover, it is also used as an antidote to insects, scorpion stings, and snakebites. The leaves’ decoction or infusion is used for the treatment of mouth diseases and toothache as a mouthwash^1,9^.

Phytochemically, seven *Haloxylon* spps. have been investigated, leading to the isolation of several alkaloids belonging mainly to seven classes, i.e., aliphatic quaternary, pyridine, indole, isoquinoline, isoquinolone, β-carboline, and phenylethylamine alkaloids^2,9^. Among 18 alkaloids, haloxynine was identified as a new piperidyl alkaloid from the aerial parts of *H. salicornicum*^10^. Also, sterols, flavonoid glycosides, and pyranones were reported in *Haloxylon* spp^11^. , which demonstrated cholinesterase and chymotrypsin inhibitory, antifungal, and nicotinergic activities. It is worth mentioning that a review article reported the presence of 107 metabolites of different classes in *Haloxylon* spp. up to 2010^12,13^. Scientists have analyzed *H. scoparium* for its phytochemical components along with biological effects. Multiple parts of the plant possess phenols together with flavonoids, followed by tannins, alkaloids, steroids, and saponins that result in its antioxidant potential and antibacterial effectiveness, as the extracted compounds from ethyl acetate demonstrated strong antibacterial properties against *Staphylococcus aureus*^8,14^. In addition, an LC-UV-MS/MS analysis showed that *H. scoparium* contains both isorhamnetin and quercetin di- and triglycosides^9^.

Liver cancer is the seventh most common cancer in the world and one of the deadliest, with a 5-year survival rate ranging from 5 to 30%. According to the Global Burden of Disease Cancer Collaboration, one in every 38 men and one in every 111 women will develop liver cancer at some stage in their lives^15,16^.

The current study aimed to investigate the *in vivo* anticancer activity of *H. scoparium* methanolic extract on hepatocellular carcinoma (HCC) by modulating the apoptotic pathway in the liver, along with the determination of its chemical profile using LC/ESI-MS/MS. Additionally, selected compounds identified in the extract were subjected to molecular docking to investigate their potential interactions with cancer- and apoptosis-related targets such as BCL-2, BAX, Caspase-3, Caspase-8, AFP, and TNF-α, to better understand their possible roles in the HCC modulation.

## Materials and methods

### Plant material

*H. scoparium* leaves were collected in January 2020 from the region of Aflou, located at the center of the Saharan Atlas (34°11′ N; 02°10′ E, 1372 m) in the Laghouat province of southern Algeria, under the relevant international guidelines and legislation, and with the necessary permissions obtained. The collection process adhered to standard practices for plant collection and preservation. The identification and authentication of the plant were carried out by Professor Mohamed KOUIDRI, Agricultural Department, Faculty of Science, Amar Telidji University, Laghouat, Algeria. A voucher specimen (No. H.s./2020) was deposited at the herbarium of the Medicinal Chemistry Department, Theodor Bilharz Research Institute, Giza, Egypt.

### Extract preparation

Dry powdered leaves of *H. scoparium* (1.2 kg) were soaked in 80% methanol (4 × 2 L) at room temperature. The combined extracts were filtered and evaporated under vacuum using a rotatory evaporator to afford a dry methanol extract of 265 g. Thereafter, it was defatted with pet. ether (60–80 °C) to afford 24.5 and 238.7 g dry samples from pet. ether-soluble portion and defatted extract, respectively.

### *In vitro* anticancer activity of *H. scoparium* extract

HepG2 cell line (ATCC: HB-8065) was provided by the Department of Cell Culture, Vacsera, Egypt. The cells were grown in a 1640 RPMI medium without PYR (Thermo Fisher Scientific, USA). The medium contained 10% FBS, 1% HEPES, and 1% antibiotic antimycotic mixture (LONZA). After attachment of the cells (7000 cells/well) in 96 tissue culture plate, different concentrations of the *H. scoparium* extract (1000, 500, 250,125, 62.5, and 31.25 µg/ml) were experienced and the plates were incubated at 37 °C in 5% CO_2_ for 24 h followed by staining using crystal violet method^17^.

### *In vivo* studies

#### Animals

The experiment was performed on male Swiss albino mice (*Musmusculus*); they were 6–8 weeks old and weighed 20 ± 15 g at the beginning of the experiment. They were provided by the animal house of Theodor Bilharz Research Institute (TBRI), Giza, Egypt. They were held in the same lab conditions of 20–25 °C, 50–60% relative humidity, and a 12-h light-dark period, with free access to normal food and water. All experiments were performed under the Institutional Ethical Committee.

#### Acute toxicity study

It was carried out to establish a safe *in vivo* dose. The extract was provided starting at 2 g/kg body weight (b.wt.) by oral gavage to mice (6 males, weight: 25–35 g, age: 6–8 weeks). For the first 4 h after dosing, the animals were monitored for toxic symptoms. Thereafter, the animals were kept for 72 h for observation of the death of mice, then maintained and observed daily for the next 13 days for any further toxicity^18,19^.

#### DEN-induced HCC in Swiss albino mice

After acclimatization for one week, forty-eight mice were randomly allocated into 4 groups (12 mice/group)^20–22^ as follows:

GpI: served as normal control and received 0.9% w/v NaCl intraperitoneally (*i.p.*).

GpII: served as the HCC-induced group, DEN was administered *i.p.* at a dose of 24 mg/kg b.wt. in 0.9% NaCl (w/v) once/week, for 12 weeks.

GpIII: was provided an oral administration of *H. scoparium* extract (200 mg/kg b.wt.) once per week for 12 weeks.

GpIV: was subjected to DEN (24 mg/kg b.wt. in 0.9% NaCl) once/week for 8 weeks, then it was administered, orally, *H. scoparium* extract (200 mg/kg) once/week for 4 weeks along with DEN (24 mg/kg b.wt. in 0.9% NaCl, *i.p*.).

### Assessed parameters for anticancer activity of *H. scoparium* in HCC-induced mice

#### Survival rate and body weight

The survival rate in each group was recorded and compared among all groups. In addition, the mice’s body weight was detected before scarifying, and its increase was compared among the animals of all groups.

#### Liver weight

At the end of the 12th week, the mice were sacrificed, and the liver was weighed to compare the relative liver weight among all groups.

#### Liver function test

The liver function test was performed using commercially available assay kits to measure serum aspartate aminotransferase (AST) and alanine transaminase (ALT) (Sclavo Diagnostics International), alkaline phosphatase (ALP) (N.S. BIO-TEC), and total direct bilirubin (TBILR) (Sclavo Diagnostics International).

#### Effect of *H. scoparium* on the apoptotic pathway in HCC-induced mice

Isolation of total RNA from serum and liver tissues was carried out using a commercially available kit (Biovision, Inc.). Quantitative PCR was performed using isolated RNA (1 µg), cDNA synthesis kit (Biovision, Inc.), and SYBR Green master mix (Thermo Fisher Scientific) to detect gene expression of tumor and apoptotic markers. A sequence of each primer was designed as in Table 1. The mean of each group was used to describe the expression ratio of each marker. The relative comparative quantitation approach was used to determine the gene expression levels (RQ = 2^^^ΔΔct).

**Table 1:** Primers sequence for gene expression analysis using qPCR.

Gene	Primer sequence	Reference
Β-actin	Forward: GGGAATGGGTCAGAAGGACT	^23^
	Reverse: CTTCTCCATGTCGTCCCAGT	
BCl-2	Forward: ATGCCTTTGTGGAACTATATGGC	^24^
	Reverse: GGTATGCACCCAGAGTGATGC	
BAX	Forward: CTACAGGGTTTCATCCAG	^25^
	Reverse: CCAGTTCATCTCCAATTCG	
Caspase-8	Forward: TTCCGGATGAGGCAGACTTT	This study
	Reverse: CCTTGTTCCTCCTGTCGTCT	
Alpha-fetoprotein (AFP)	Forward: CTACATTTCGCTGCGTCCAA	This study
	Reverse: CAGCCAACACATCGCTAGTC	
Tumor necrosis factor-α (TNF-α)	Forward: ACCCTCACACTCACAAACCA	^26^
	Reverse: GGCAGAGAGGAGGTTGACTT	

### Liver histopathology

The isolated livers were fixed in 10% buffered formalin for histopathological investigations. Liver pieces were processed routinely into paraffin blocks. A total of 4–5 μm thick sections were cut on positively charged glass slides. Sections were then stained with hematoxylin and eosin stain (H&E) for light microscopic histopathological examination regarding the hepatic architecture, inflammation, dysplasia, and malignancy. Masson trichrome stain was used for the assessment of tissue fibrosis. The liver histology from different groups was compared using an Axio microscope (Zeiss), and photos were taken using the attached digital Mrc5 camera (Zeiss).

### Immunohistochemistry of caspase-3

Deparaffinizing and rehydrating the tissue pieces were done by immersing the slides in xylene with two changes for 10 min. each, followed by rehydration of tissue sections by immersing the slides in decreasing grades of ethanol. The slides were placed in a microwave-safe tray with 10 mM sodium citrate buffer (pH 6.0) and 0.05% Tween 20 to achieve antigen retrieval. The slides were then microwaved for 5 min., while being kept at a temperature just below boiling. The cooling process took 30 min. at room temperature. The slides were immersed in TBST 3 times, 3 min. each, to wash them (Tris Buffered Saline having 0.05% Tween 20). The slides were incubated in 3% hydrogen peroxide produced in methanol for 15 min., while they were kept in the dark to quench endogenous peroxidase. Following three TBST washes on each slide for 3 min. each, a 1:100 dilution of the Caspase-3 antibody (Rabbit anti-Caspase-3 antibody, Abcam Cat no. ab184787), in one volume of PBS, was utilized. At 4 °C, in a humid environment, sections were incubated with diluted primary antibodies overnight. According to the manufacturer’s recommendations, the sections were first washed 4 times, for 5 min. each, with TBST, followed by 30 min. of incubation with the secondary biotinylated antibody (Biotinylated Goat Anti-Rabbit IgG Abcam Cat no. ab64256) and another 30 min. with avidin-peroxidase complex. The expression level of Caspase-3 in tissue cells was judged according to the percentage of caspase-3 positive cells in each sample. Specifically, a percentage of ≤ 10% was judged negative and > 10% was positive^27,28^.

All sections were assessed and scored. The sections were examined by using a light microscope [Scope A1, Axio, Zeiss, Germany]. Photomicrographs were taken using a microscope camera [AxioCam, MRc5, Zeiss, Germany].

### LC/ESI-MS/MS analysis

The chemical composition of *H. scoparium* leaf extract was performed using the LC/ESI-MS/MS technique in negative ion mode according to the reported procedures^29,30^.

### *In silico* studies

Several molecular targets play critical roles in the progression and regulation of hepatocellular carcinoma (HCC), making them valuable candidates for *in silico* docking studies. B-cell lymphoma 2 (Bcl-2) is an anti-apoptotic protein often overexpressed in HCC, contributing to tumor survival by inhibiting mitochondrial-mediated apoptosis^31^. In contrast, BAX, a pro-apoptotic member of the Bcl-2 family, promotes programmed cell death and is typically downregulated in liver cancer, thus aiding tumor evasion of apoptosis^32^. Caspase-8 functions as an initiator of the extrinsic apoptotic pathway and is frequently suppressed in HCC, facilitating immune escape and cell proliferation^33^. Caspase-3, an executioner caspase, plays a crucial role in the terminal phase of apoptosis; its reduced activity in HCC is associated with enhanced tumor survival and chemoresistance^34,35^. Alpha-fetoprotein (AFP) is a well-established HCC biomarker; beyond its diagnostic value, it is implicated in cell growth regulation, immune suppression, and oncogenic signaling^36^. Additionally, tumor necrosis factor-alpha (TNF-α), a central pro-inflammatory cytokine, contributes to liver inflammation, fibrosis, and carcinogenesis and exhibits a complex dual role in both tumor suppression and promotion depending on the tumor microenvironment^36^. These targets collectively represent key nodes in the apoptotic and inflammatory pathways dysregulated in HCC and thus are critical for computational docking and therapeutic exploration.

#### Ligand preparation

The study focused on bioactive compounds identified from *H. scoparium*, a plant known for its ethnomedicinal applications. All phytocompounds were selected, and control sorafenib was acquired from ChemDraw and the PubChem database (https://pubchem.ncbi.nlm.nih.gov/) in the form of 3D SDF format and subsequently converted to PDB format using Open Babel GUI software, ensuring compatibility with docking tools.

#### Target protein selection

Key apoptosis and cancer regulation proteins were selected as targets: Bcl-2 PDB ID:2W3L, BAX PDB ID:4S0O, Caspase-3 PDB ID:3GJQ, Caspase-8 PDB ID:4PRZ, AFP PDB ID:7YIM, and TNF-α PDB ID:2AZ5. These targets were chosen based on their established roles in cell survival and death signaling pathways and downloaded in the PDB file format from the RCSB Protein Data Bank (https://www.rcsb.org/).

#### Molecular docking

For docking, the proteins must be prepared by eliminating all water molecules, heteroatoms, and any Co-crystallized inhibitors. Additionally, hydrogen atoms and Kollman charges were added to the receptors using Autodock tools (ADT) (version 1.5.6). The docking process was performed using the AutoDock Vina program^37^. The grid box was set to cover the entire active site of each protein. The best binding poses were selected based on binding energy (kcal/mol).

#### Visualization and analysis

Docked complexes were visualized using UCSF ChimeraX 1.9 and BIOVIA Discovery Studio Visualizer v 21, which facilitated both 3D and 2D interaction analysis. Box plots illustrating binding energy distributions were generated using OriginPro 2025.

### Statistical analysis

Data in the treatment groups were presented as mean ± SEM, and statistical analysis was performed using GraphPad Prism 8. One-way or Two-way ANOVA was followed by post hoc Tukey’s multiple comparison tests. It was determined that the *P* < 0.05 was statistically significant.

## Results

### *In vitro* cytotoxicity of *H. scoparium* against the HepG2 cell line

The calculated IC_50_ was 282.4 ± 35.88 µg/ml. A statistical significance was detected among different concentrations (1000, 500, 250, 125, 62.5, and 31.25 µg/ml), using One-way ANOVA (*p* < 0.0001).

### Acute toxicity study

There was no mortality or apparent toxicity from a dosage of 2 g/kg b.wt., such as diarrhea, urination, edema, or skin color change. Also, after 14 days of observation, there was no mortality in these mice. On a dosage of 200 mg/kg b.wt. orally, the extract was considered to be non-toxic based on these observations and the literature.

### Assessed parameters for anticancer activity of *H. scoparium* in HCC-induced mice

#### Survival rate and body weight

**Fig. 1:**
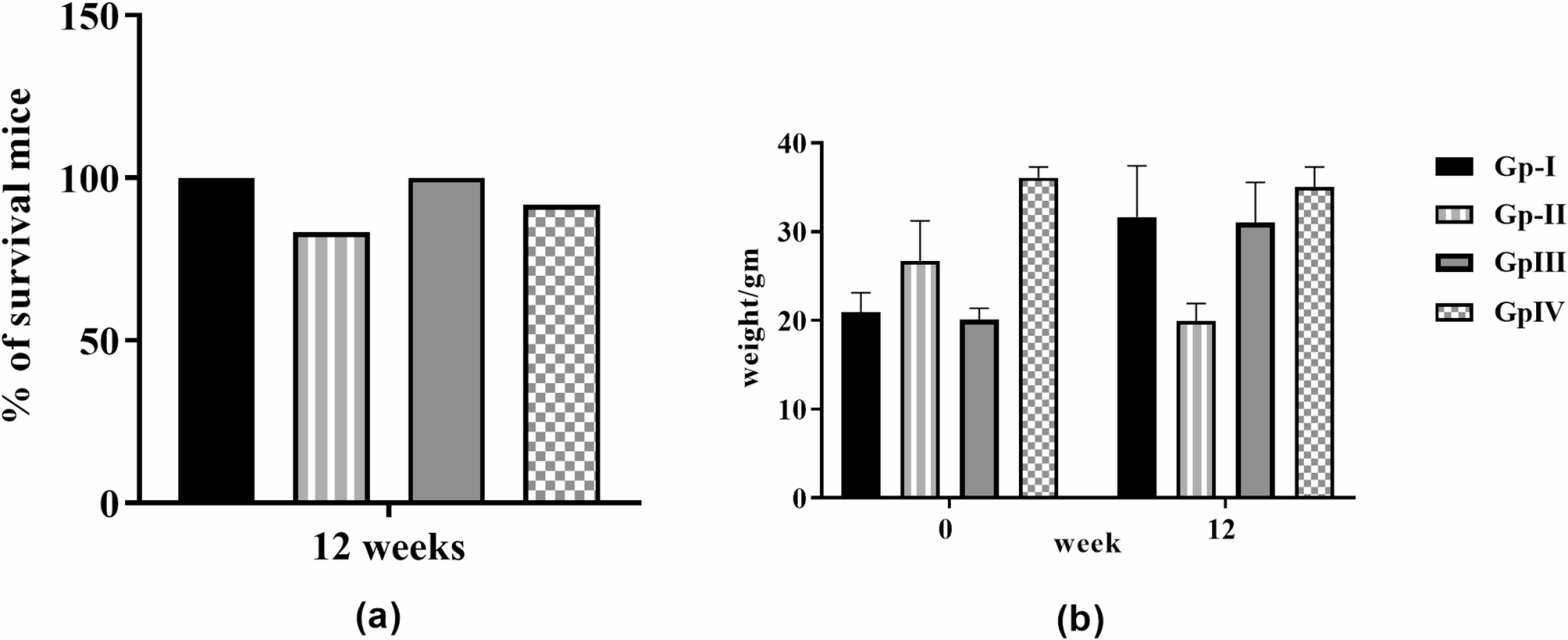
(**a**) Percent of surviving mice at the end of the experiment (12 weeks). Results showed that *H. scoparium* post-treatment protected mice from death, and its dose was safe, as found in the *H. scoparium* group. (**b**) Effect of the *H. scoparium* extract on the b. w. of mice with HCC induced by DEN.

The survival of mice in each group was monitored throughout the experiment. Results showed that no death was found in both normal and *H. scoparium* groups. Nevertheless, 83.33 and 91.67% of mice survived in DEN and DEN/*H. scoparium* groups, respectively (Fig. 1a). In Table 2; Fig. 1b, the variation in the b.wt. was recorded among all groups at the beginning of the experiment (0 weeks) and before sacrificing (12 weeks). There was a significant reduction in the b.wt. in the DEN group compared to the normal group. Furthermore, the b.wt. increased significantly in all groups, and the *H. scoparium* extract did not prevent this increase, showing a pattern similar to the normal group. Besides, post-treatment in DEN/*H. scoparium* group showed that the extract significantly prevented the loss of the b.wt. compared to the normal and DEN groups (*p* < 0.0001).

**Table 2:** Body weight of mice in each group.

Week	Normal	DEN	*H. scoparium*	DEN/*H. scoparium*
0	20.96 ± 0.62	26.69 ± 0.43	20.07 ± 1.3	36.07 ± 0.35
12	31.65 ± 1.66	19.9 ± 0.64	31.05 ± 1.36	35.06 ± 0.67

#### Liver weight

Analysis using One-way ANOVA followed by hoc Tukey’s multiple comparison tests showed that there was a statistical significance between normal and DEN groups (*p* = 0.0034) and between DEN and DEN/*H. scoparium* groups (*p* = 0.045). However, there was no significant difference between *H. scoparium* and DEN/*H. scoparium* or normal and *H. scoparium* (Table 3).

**Table 3:** Liver weight of mice in each group.

Week	Normal	DEN	*H. scoparium*	DEN/*H. scoparium*
12	1.42 ± 0.43	1.06 ± 0.31	1.18 ± 0.23	1.5 ± 0.37

#### Liver function test

**Fig. 2:**
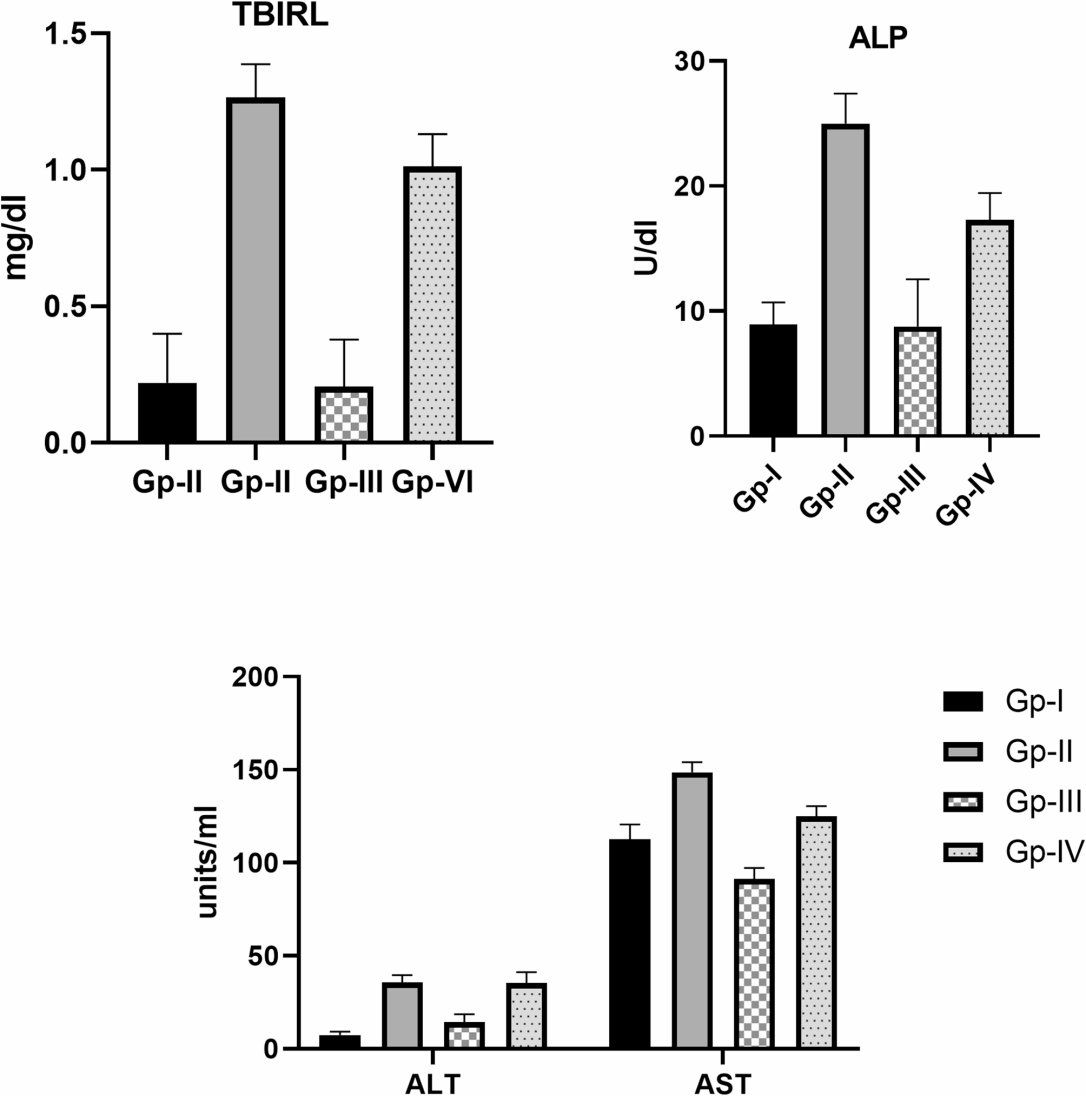
Effect of the *H. scoparium* extract on the liver function in DEN-induced HCC in mice.

There was a significant increase in the level of AST, ALT, and ALP in the DEN group compared to the normal group, and no significant difference in TBILR was recorded. In the *H. scoparium* group, a significant difference was observed in the level of AST and ALT compared to both normal and DEN groups. Moreover, findings referred to a significant decrease in TBILR, ALP, and AST levels of DEN/*H. scoparium* group compared to the DEN group (Fig. 2).

### Effect of *H. scoparium* on the apoptotic pathway in HCC-induced mice

**Fig. 3:**
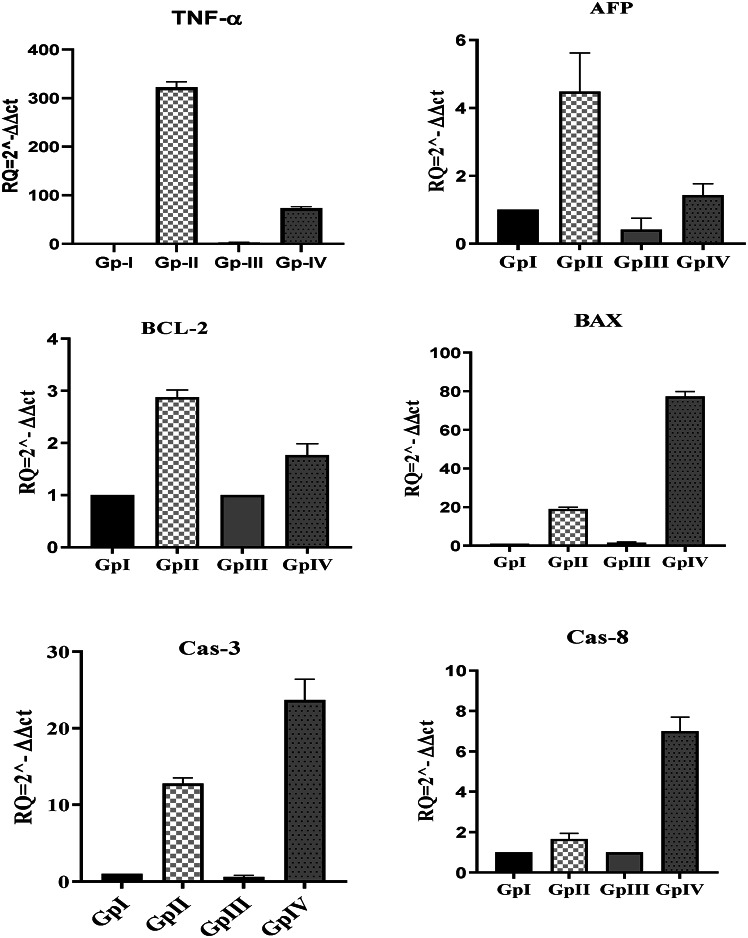
Relative quantification of inflammatory marker (TNF-α), tumor markers (AFP and BCl-2), and apoptotic markers (BAX, Caspase-3, and Cas-8) in HCC-induced mice.

The relative expression of serum TNF-α was significantly increased in the DEN group (322.44 ± 11.2) compared to the normal group (0.07 ± 0.02). In the *H. scoparium* group, the relative expression was 2.48 ± 0.6. However, it was significantly decreased in the DEN/*H. scoparium* group (73.98 ± 2.7) compared to the DEN group. The relative expression level of tumor markers Bcl-2 and AFP was significantly increased in the DEN group compared to the other groups. In addition, apoptotic markers BAX, Caspase-3, and Cas-8 were significantly increased in the DEN/*H. scoparium*-treated group compared to the normal and DEN groups (Fig. 3).

### Effect of *H. scoparium* extract on the treatment of HCC supported by histopathology and immunohistochemistry

**Fig. 4:**
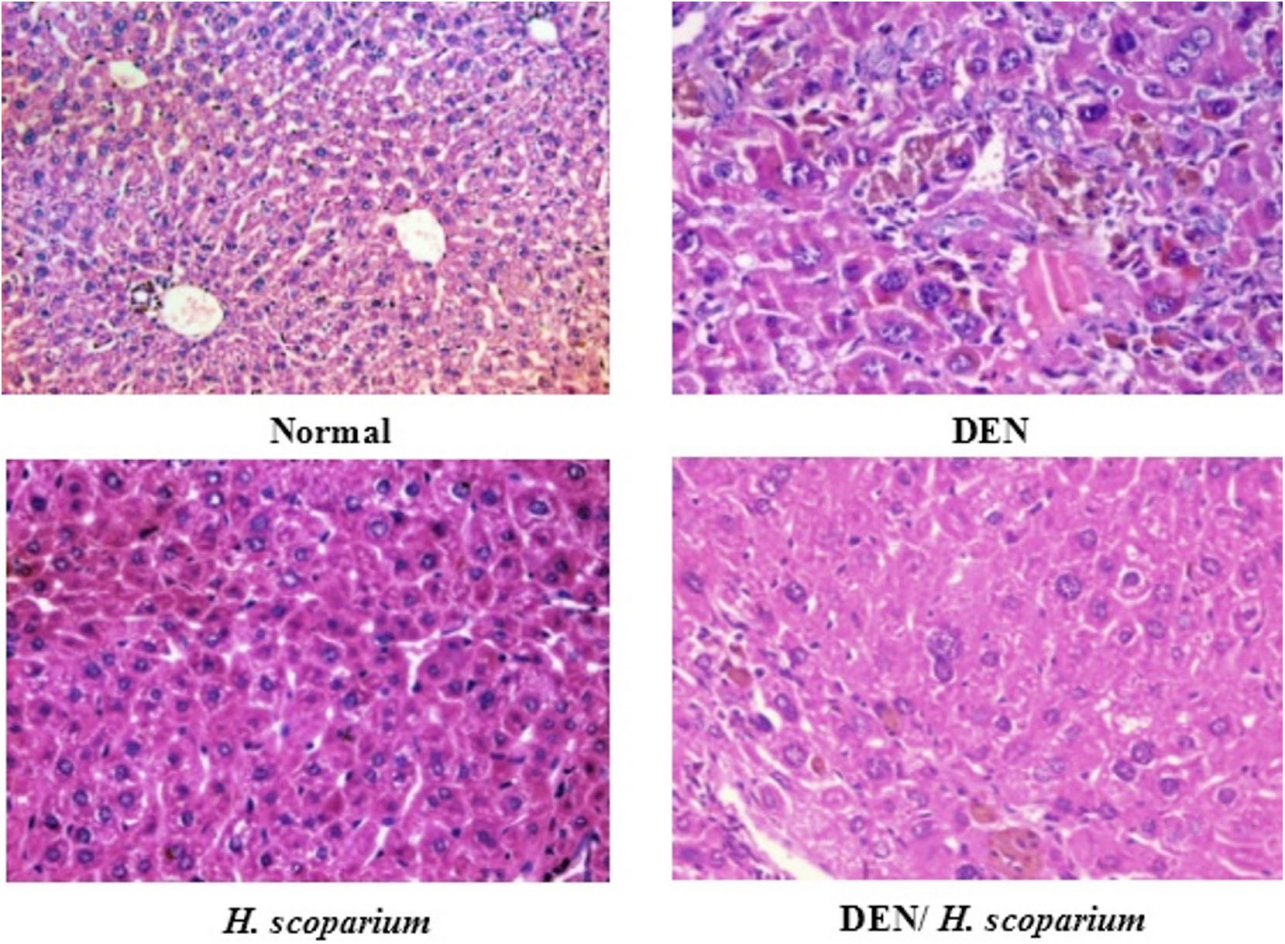
Histopathology of the liver isolated from mice with DEN-induced HCC.

**Fig. 5:**
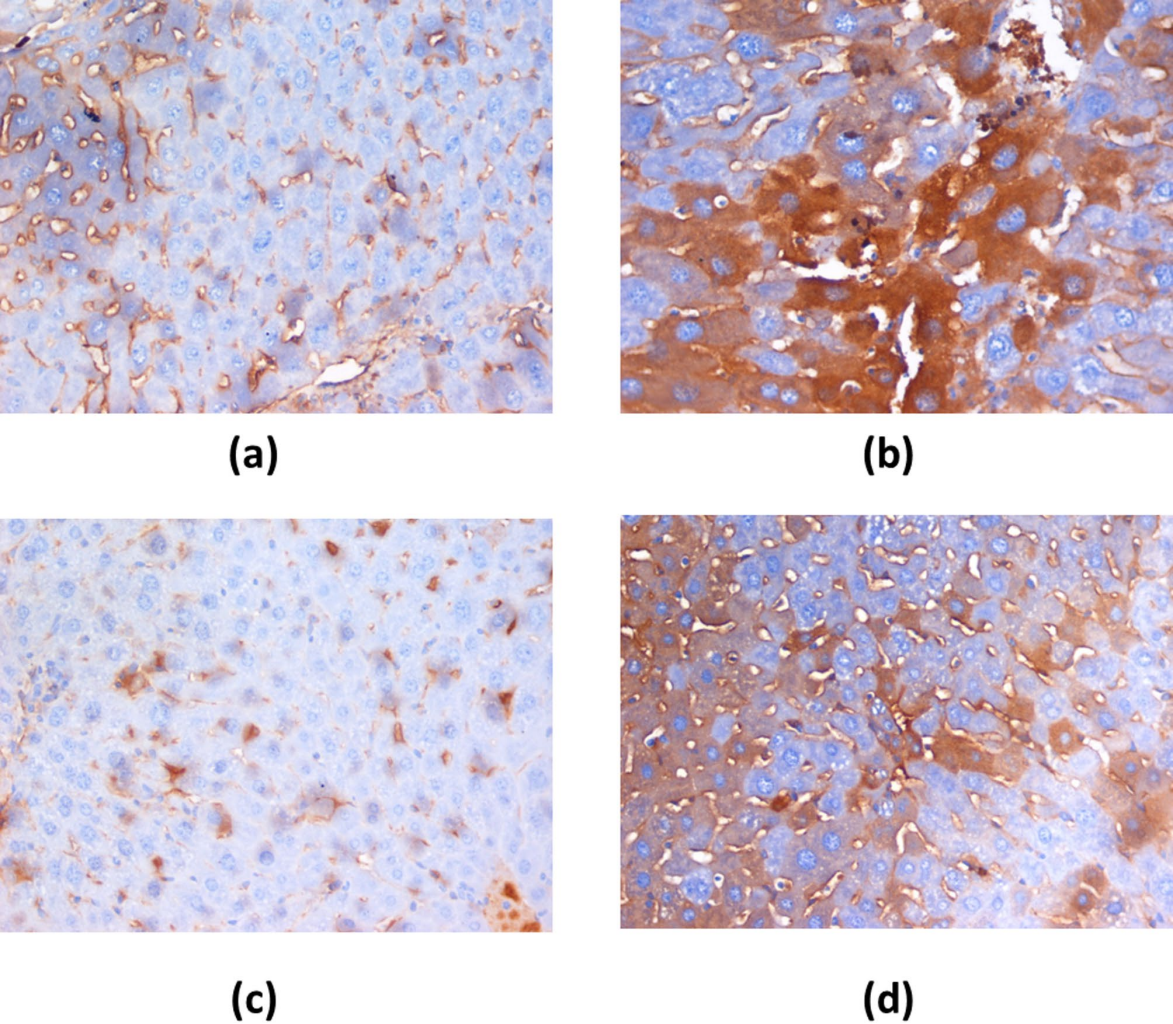
Immunohistochemistry of Caspase-3 protein detection (X400). (**a**) Section in the liver of Normal mice showing predominantly sinusoidal expression of Caspase-3 with moderate intensity. (**b**) Section in the liver of DEN-treated mice showing remarkable dense expression of Caspase-3 within hepatocytes (brownish discoloration). (**c**) Section in the liver of HS-treated mice showing focal mild expression of Caspase-3 within hepatocytes and sinusoids. (**d**): Section in the liver of HS + DEN-treated mice showing increased dense expression of Caspase-3 within hepatocytes.

Histopathology is depicted in a photograph (Fig. 4). Hepatocytes (with granular cytoplasm that took up the acidophilic stain) and nuclei (centrally located) were seen in the histology of both normal and *H. scoparium* groups. With H&E staining, the central vein and bile ducts can be seen clearly. Together with areas of necrosis, cholestasis, bile duct proliferation, and lymphatic dilatation, a distorted architecture, focal HCC, and dysplasia are seen in the histology of the DEN group. However, the hepatic architecture is more preserved with minor hepatocytic changes in the post-treated DEN/*H. scoparium* group, but with scattered foci of hepatocytic apoptosis. On the other hand, the *H. scoparium*-treated group showed more or less normal hepatic architecture with no histopathological changes. Results of IHC showed a mild expression of Caspase-3 in both normal and *H. scoparium* groups. However, an increased expression of Caspase-3 was detected in both DEN and DEN/*H. scoparium* groups as shown in Fig. 5; Table 4.

The normal group shows a preserved hepatic lobular architecture with the absence of degeneration, inflammation, or fibrosis. DEN group shows HCC with focal acinar formation and bizarre-shaped hyperchromatic nuclei with focal cholestasis and distorted lobular pattern. *H. scoparium* group shows preserved hepatic lobular architecture with focal cholestasis and mild hepatocytic hydropic changes. DEN/*H. scoparium* group shows focal dysplasia, few apoptotic figures, and cholestasis.

**Table 4:** Caspase-3 Immunohistological expression in each group.

	Normal GpI	DEN GpII	*H. scoparium* GpIII	DEN/*H. scoparium* GpIV
Percentage (M ± SD)	1.05 ± 0.33	22.87 ± 5.16	3.04 ± 0.24	15.36 ± 4.55
Density score	++	+++	++	+++

### Total phenolic (TPC) and total flavonoid (TFC) contents

As illustrated in the Table 5, the *H. scoparium* leaf extract exhibited substantial total phenolic (TPC) and total flavonoid (TFC) contents when examined using the Folin–Ciocalteu’s and aluminum chloride assays, respectively. The TPC was determined to be 157.67 ± 4.84 mg GAE/g dry extract. Also, the TFC was determined to be 65.57 ± 1.89 mg RE/g dry extract.

**Table 5:** Total phenolic (TPC) and total flavonoid (TFC) contents of the *H. scoparium* leaf extract.

Tested extract	TPC (mg GAE/g dry extract)	TFC (mg RE/g dry extract)
*H. scoparium*	157.67 ± 4.84	65.57 ± 1.89

Data are presented as mean ± S.D., *n* = 3. GAE: gallic acid equivalent; RE: rutin equivalent.

### Chemical profiling of the *H. scoparium* leaf extract

**Fig. 6:**
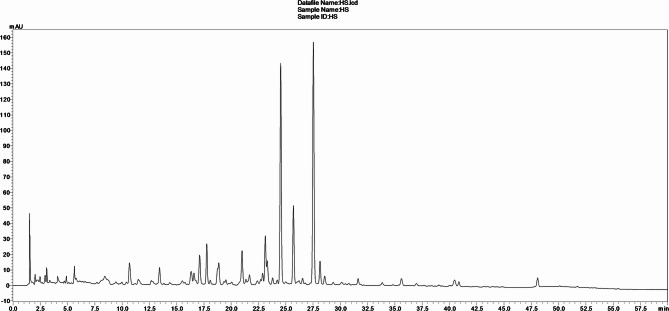
TIC chromatogram from LC/ESI-MS/MS analysis of *H. scoparium* leaf extract.

The LC/ESI-MS/MS examination of *H. scoparium* leaf extract furnished 27 compounds comprising organic acids, phenolic acid derivatives, and flavonoids (Fig. 6; Table 6). Interestingly, two organic acids were annotated in the tested extract. They furnished [M‒H]^‒^ anions at *m/z* 191, and 133, and daughter ions at 111 [M‒H‒80]^‒^, and 115 [M‒H‒18(H_2_O)]^‒^; they were assigned as citric, and malic acids, respectively. A characteristic set of gallic acid derivatives was observed in the tested extract. They disclosed their own molecular ions ([M‒H]^‒^) at *m/z* 331, 463, 301, and 343, and daughter ions at 169 [M‒H‒162(Glu)]^‒^, 331 [M‒H‒132(pentose)]^‒^, 169 [M‒H‒132(pentose)]^‒^, and 191 [M-H‒152(galloyl moiety)]^‒^. They were assigned as galloylglucose, galloyl-pentosylglucose, galloylpentose, and galloylquinic acid, respectively. As well, another set of gentisic acid derivatives was also detected in the tested extract. They exhibited molecular ions [M‒H]^‒^ at *m/z* 315, 329, 285, and 417, with identical daughter ions at 153 [M‒H‒162(Glu)]^‒^, 153 [M‒H‒176(Gluc)]^‒^, 153 [M‒H‒132(pentose)]^‒^, and 153 [M‒H‒264(2 pentose)]^‒^. They were identified as the glucoside, glucuronide, pentoside, and dipentoside of gentisic acid, respectively. Another peak (R_*t*_= 13.11) demonstrated a molecular ion at *m/z* 153 [M‒H]^‒^ and a decarboxy daughter ion at 109 [M‒H‒44(CO_2_)]^‒^ that was interpreted as gentisic acid. Three signals demonstrated their molecular ions ([M‒H]^‒^) at *m/z* 261, 369, and 515 and daughter ions at 181 [M‒H‒80(sulfate moiety)]^‒^, 193 [M‒H‒176]^‒^, and 191 [M‒H‒324(2 caffeoyl moiety)]^‒^ that were tentatively identified as dihydrocaffeic acid sulphate, ferulic acid 4-*O*-glucuronide, and dicaffeoylquinic acid, respectively. Moreover, a set of isorhamnetin glycosides was annotated in the tested extract. They furnished deprotonated ions ([M‒H]^‒^) at *m/z* 917, 725, 755, 623, and 477, with identical aglycone ions at 315 [M‒H‒602]^‒^, 315 [M‒H‒410]^‒^, 315 [M‒H‒440]^‒^, 315 [M‒H‒308(rutinoside moiety)]^‒^, and 315 [M‒H‒162(Glu)]^‒^. In addition their spectra disclosed also two characteristic daughter ions at 314 and 299 amu that were interpretable for the common online oxidation of the previous aglycone fragment and demethylation, respectively. Accordingly, they were assigned as isorhamnetin-*O*-hydroxyferuloylhexoside-*O*-malonylhexoside, malonyldiglucoside, triglycoside, rutinoside, and glucoside of isorhamnetin, respectively. The other four flavonoid derivatives were picked up from the TIC of the tested extract (R_*t*_= 16.33, 20.98, 24.53, 34.81 min.). They disclosed [M‒H]^‒^ ions at *m/z* 785, 741, 741 and 431 with daughter ions at 315 [M‒H‒470]^‒^, 301 [M‒H‒440]^‒^, 301 [M‒H‒440]^‒^, and 269 [M‒H‒162]^‒^, assignable for isorhamnetin glycoside, two quercetin triglycosides, and an apigenin glucoside.

**Table 6:** Chemical composition of the *H. scoparium* leaf extract by LC/ESI-MS/MS analysis.

R_*t*_	[M-H]^−^	MS/MS	Tentative identification	Ref.
1.45	191	173, 129, 111, 87, 57	Citric acid	^38^
1.57	133	115, 89, 71	Malic acid	^39^
3.68	331	313, 271, 169, 125	Galloylglucose	^40^
4.39	331	313, 271, 169, 125	Galloylglucose	^40^
5.06	315	153, 109, 81, 42	Gentisic acid glucoside	^41^
5.12	329	153, 109, 81, 42	Gentisic acid glucuronide	^42^
5.72	463	331, 169, 151, 125	Galloyl-pentosyl glucose	^43^
5.84	285	153, 109, 108	Gentisic acid pentoside	^44^
6.44	301	169, 125	Galloylpentose	^45^
6.56	261	181, 179, 137, 109	Dihydrocaffeic acid sulphate	^46^
7.70	343	191, 169, 125, 111, 107	Galloylquinic acid	^47^
8.18	285	153, 109, 108	Gentisic acid pentoside	^44^
10.67	417	285, 241, 153, 109, 108	Gentisic acid dipentoside	^48,49^
13.11	153	135, 123, 109, 108	Gentisic acid	^50^
13.42	369	193, 178, 149, 134, 119	Ferulic acid 4-*O*-glucuronide	^51^
16.33	785	639, 623, 459, 315, 300, 271, 151	Isorhamnetin 3-*O*-rutinoside-7-glucoside	^52^
17.68	917	873, 669, 505, 315, 314, 299	Isorhamnetin-*O*-hydroxyferuloylhexoside-*O*-malonylhexoside (isomer 1)	^52^
18.85	917	873, 669, 505, 315, 314, 299	Isorhamnetin-*O*-hydroxyferuloylhexoside-*O*-malonylhexoside (isomer 2)	^52^
20.98	741	609, 595, 301, 300, 255, 179, 151	Quercetin-*O*-pentoside-*O*-deoxyhexoside-hexoside	^52,53^
23.12	725	563, 315, 314, 299	Isorhamnetin malonyldiglucoside	^54^
24.53	741	609, 595, 301, 300, 255, 179, 151	Quercetin-*O*-pentoside-*O*-deoxyhexoside-hexoside	^52,53^
25.69	755	623, 461, 315, 314, 300, 299, 255, 179, 151	Isorhamnetin-*O*-deoxyhexosyl-pentosyl-hexoside	^55,56^
27.52	623	477, 461, 315, 314, 300, 299, 255, 179	Isorhamnetin-*O*-rutinoside (isomer 1)	^57^
28.13	623	477, 461, 315, 314, 300, 299, 255, 179	Isorhamnetin-*O*-rutinoside (isomer 2)	^57^
28.50	477	315, 314, 300, 299, 255, 179, 151	Isorhamnetin 3-*O*-glucoside	^58^
30.71	515	353, 335, 191, 179, 173, 151, 135	Dicaffeoylquinic acid	^29,59^
34.81	431	311, 269, 225	Apigenin 7-*O*-glucoside	^60^

### Docking scores and best binding interactions

The molecular docking results provide strong evidence that *H. scoparium*-derived flavonoid glycosides, especially quercetin and isorhamnetin glycosides (Table 7), exhibit high binding affinities toward multiple apoptosis and cancer-related targets. Quercetin triglycoside showed the most robust interaction profile, particularly with BCL-2 (-10.5 kcal/mol), BAX (-10.2 kcal/mol), Caspase-8 (-10.5 kcal/mol), and TNF-α (− 10.4 kcal/mol), suggesting its potential as a broad-spectrum inhibitor of both pro- and anti-apoptotic proteins. Similarly, isorhamnetin triglycoside showed exceptional affinity for TNF-α (− 10.6 kcal/mol), making it a promising candidate for modulating inflammatory signaling.

**Fig. 7:**
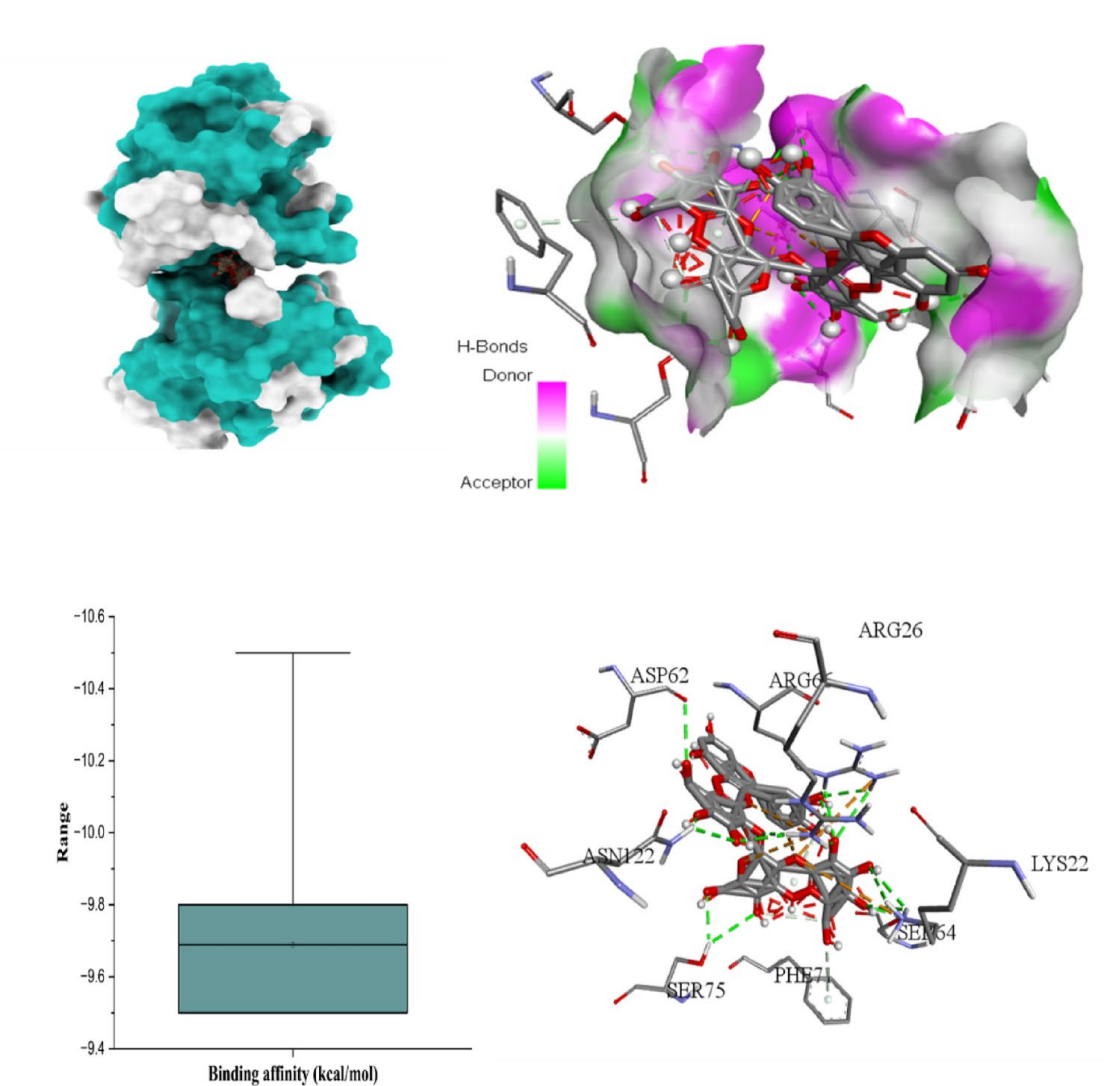
3D binding interaction and Box plot representing the docking score (binding affinity) in kcal/mol of Quercetin triglycoside with the BCL-2 protein.

**Fig. 8:**
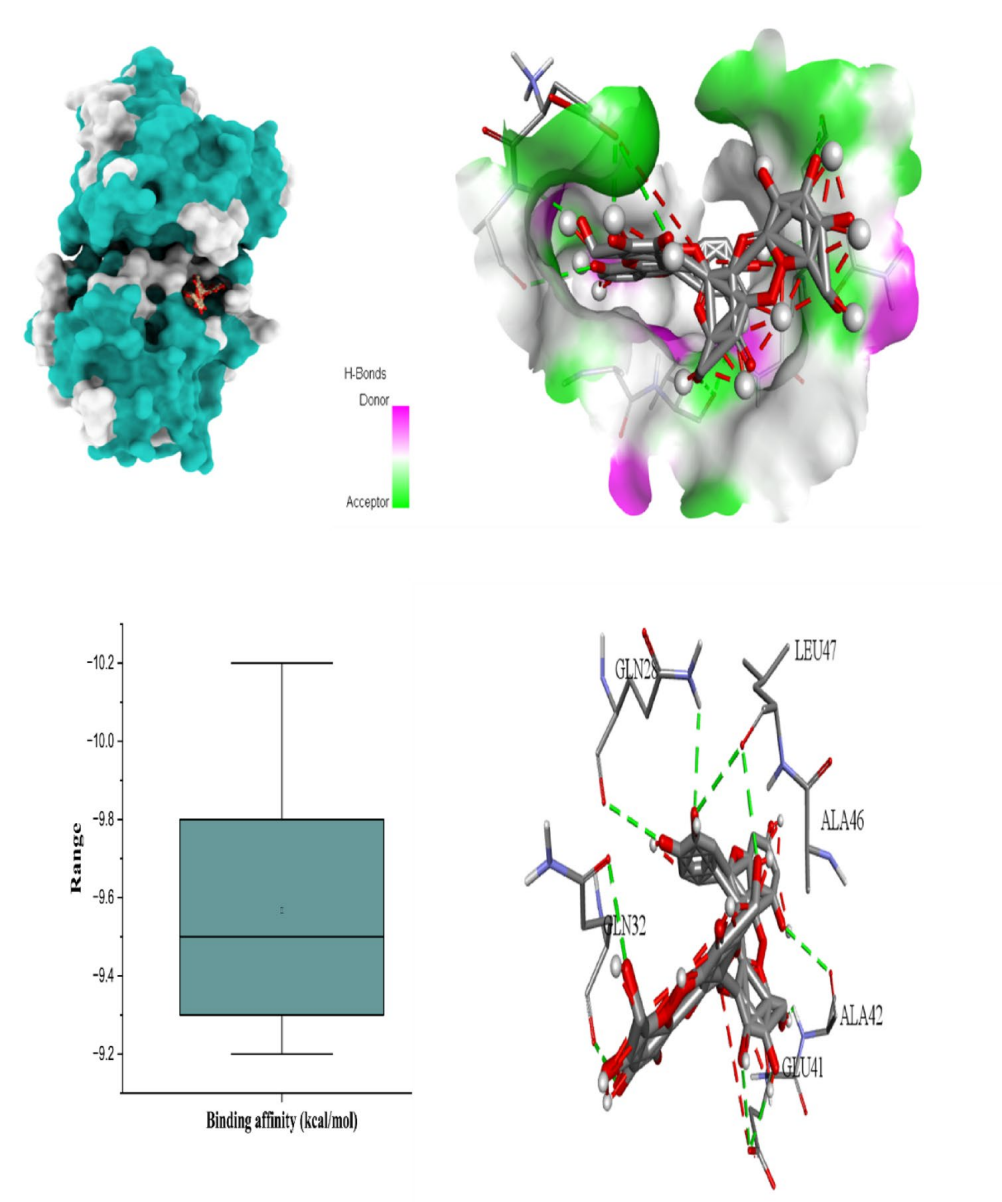
3D binding interaction and Box plot of Quercetin triglycoside with the BAX protein.

**Fig. 9:**
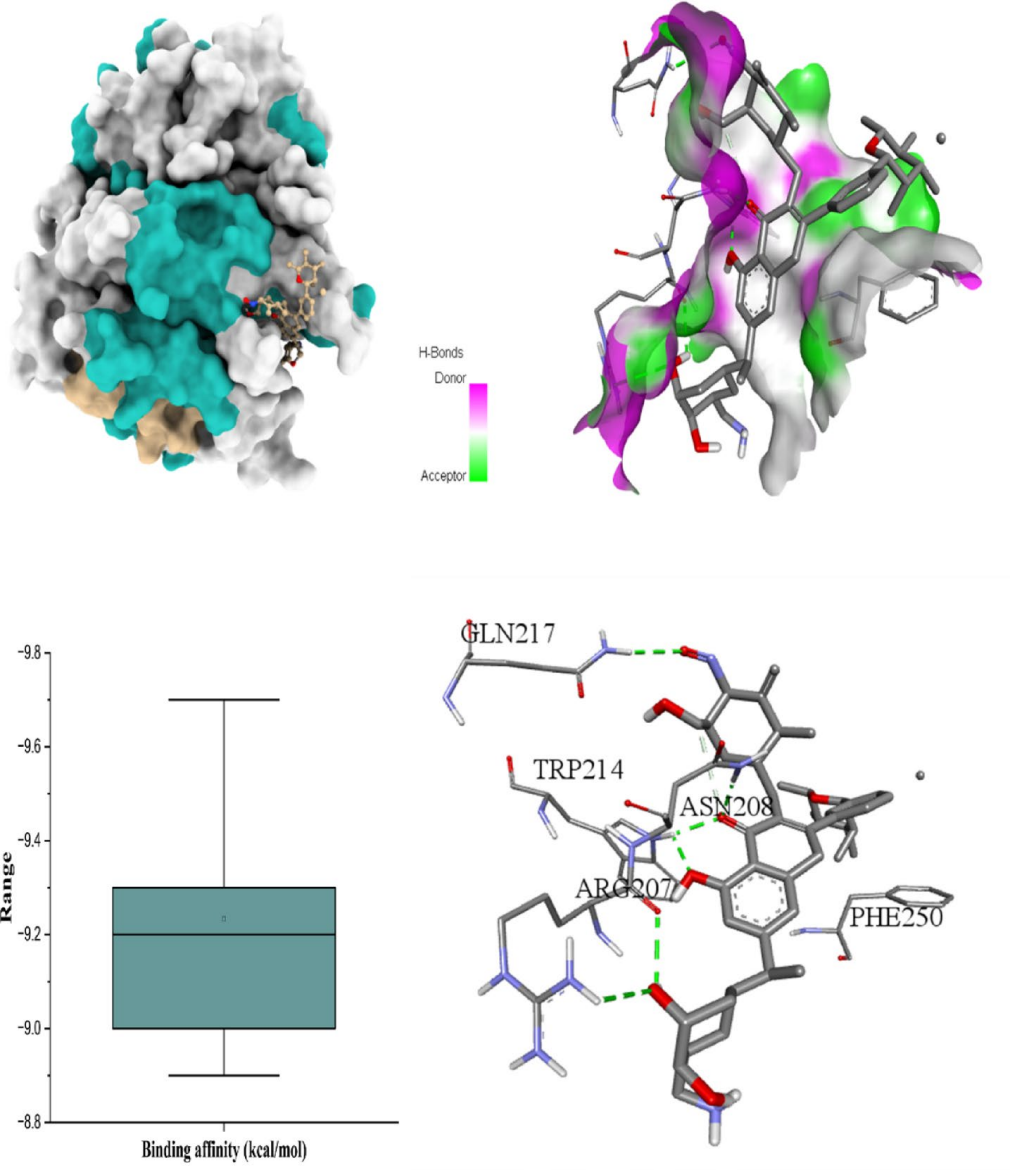
3D binding interaction and Box plot of Isorhamnetin triglycoside with Caspase-3.

**Fig. 10:**
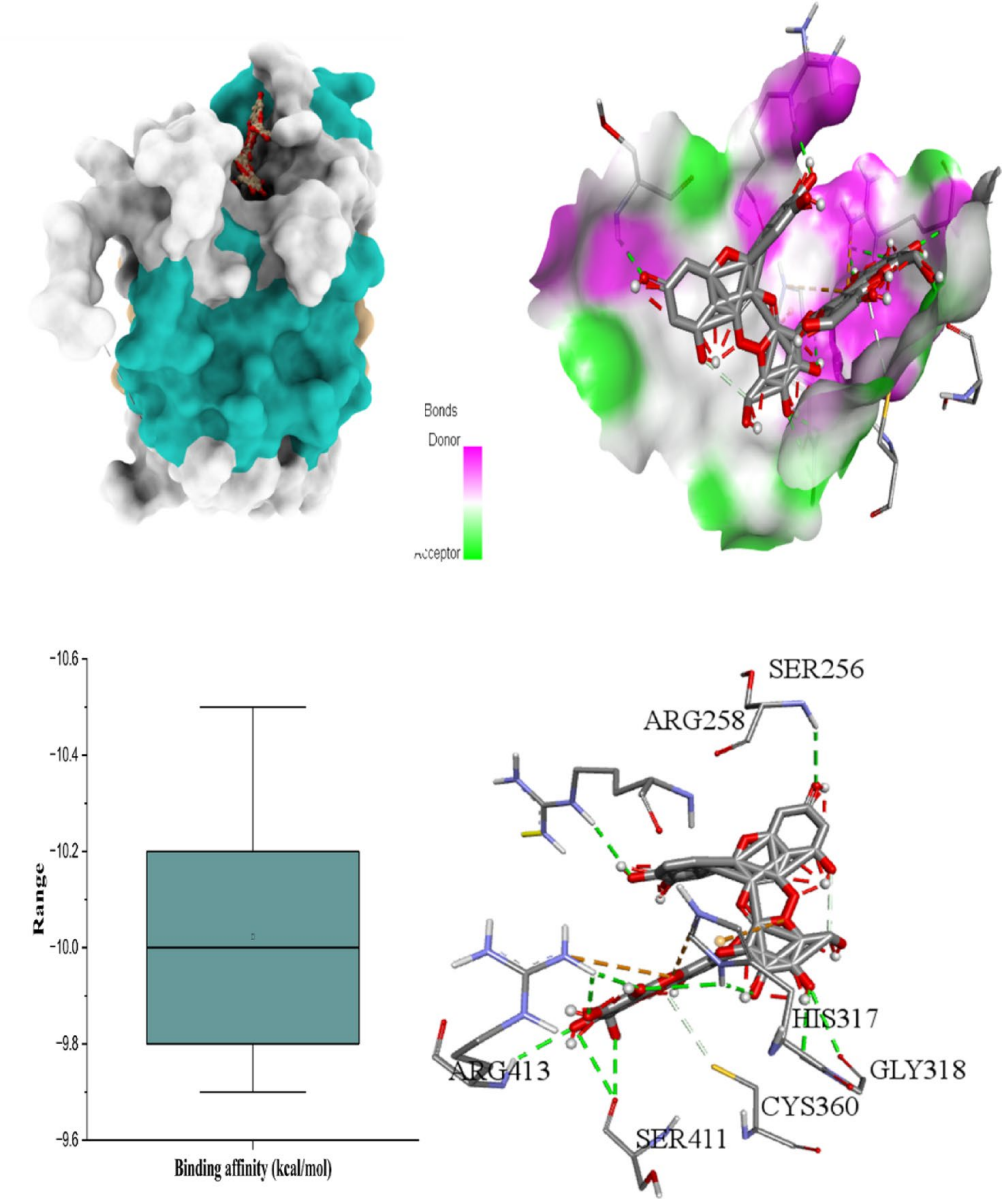
3D binding interaction and Box plot of Quercetin triglycoside with Caspase-8.

**Fig. 11:**
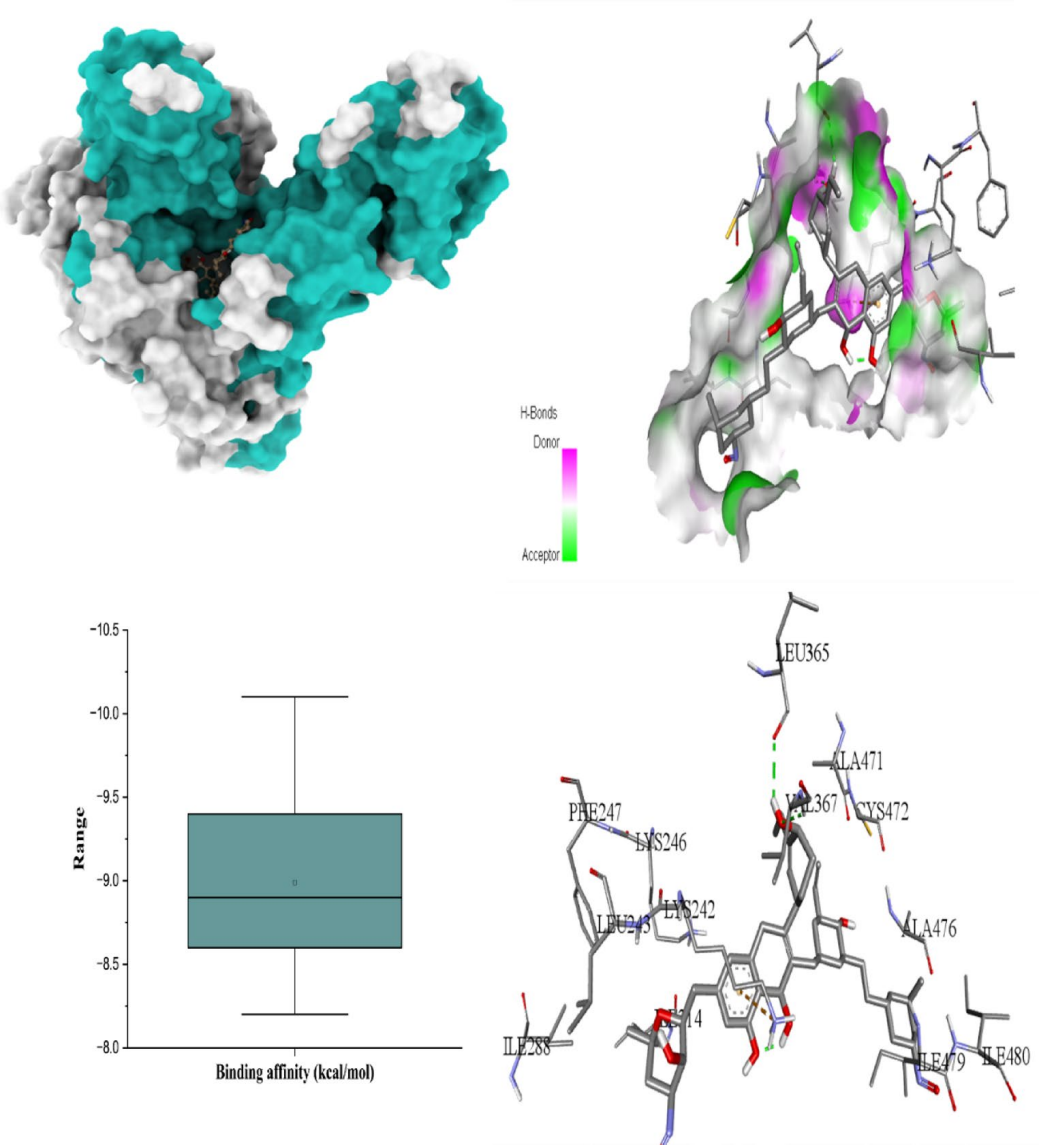
3D binding interaction and Box plot of Isorhamnetin triglycoside with AFP.

**Fig. 12:**
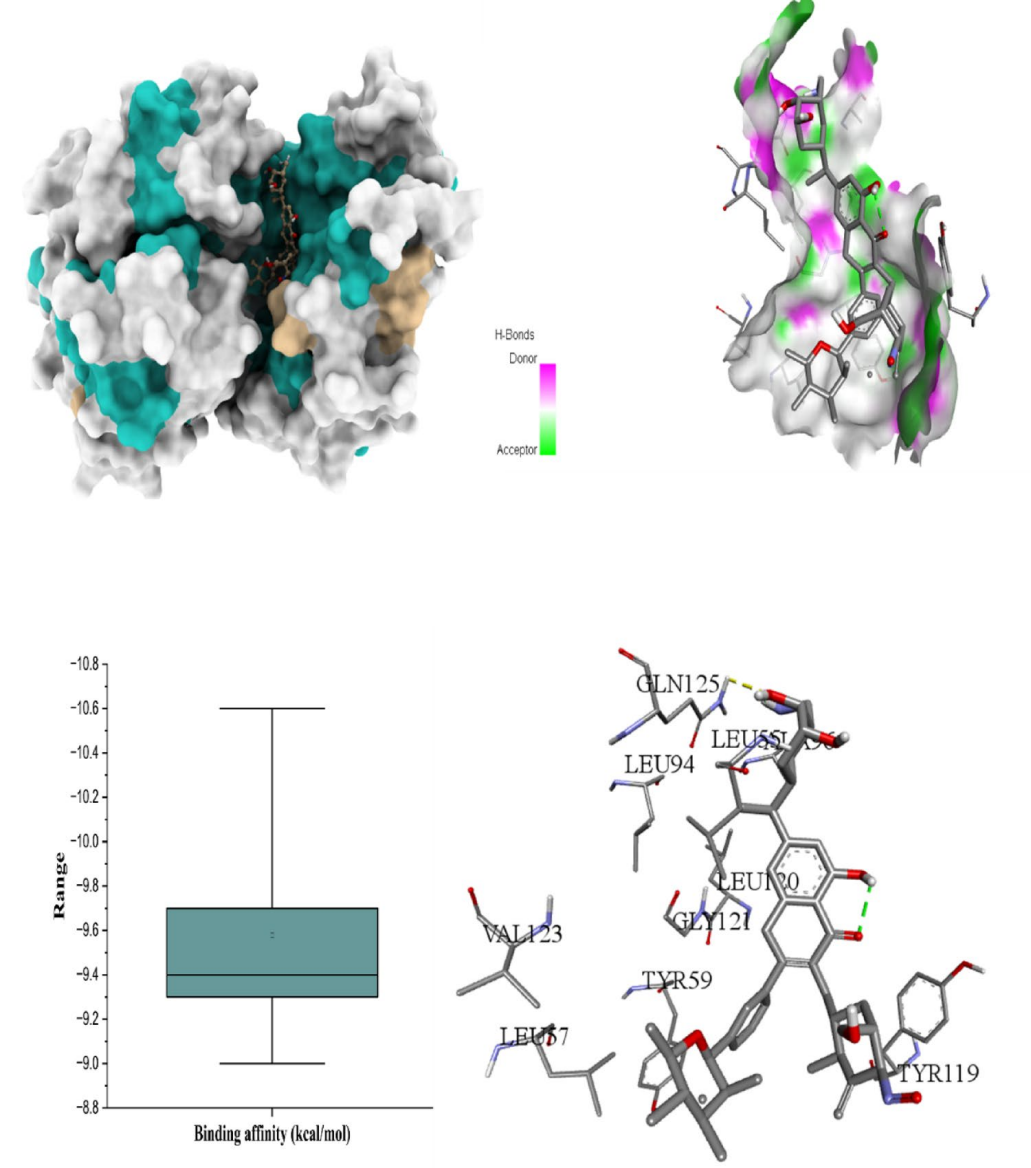
3D binding interaction and Box plot of Isorhamnetin triglycoside with TNF-α.

The six figures illustrate the molecular docking interactions of potent *H. scoparium* compounds with key cancer- and apoptosis-related targets, highlighting both binding affinity and specific residue interactions. Figure 7 shows quercetin triglycoside bound to BCL-2, where the ligand forms hydrogen bonds with residues such as LYS22 (2.73Å), ARG26 (3.35 Å), SER64 (3.11 Å), ARG66 (1.97 Å), ASP62 (2.93 Å), SER75 (2.29 Å), and ASN122 (2.10 Å). Figure 8 depicts its binding to BAX, engaging GLN28 (2.54Å), GLN32 (3.08Å), GLU41 (2.73Å), ALA42 (3.30Å), and LEU47(4.02), suggesting the potential to influence BAX-mediated apoptotic signaling. Figure 9 presents isorhamnetin triglycoside interacting with Caspase-3, anchored by hydrogen bonds and π–π stacking with residues such as GLN217(2.18 Å), TRP214 (2.08 Å), ASN208 (2.13 Å), ARG207 (2.78 Å), and PHE250 (4.74 Å), possibly enhancing caspase activation. In Fig. 10, quercetin triglycoside exhibits strong interaction with Caspase-8, forming key bonds with residues like SER256 (2.46 Å), ARG258 (2.62 Å), HIS317 (2.38 Å), GLY 318 (2.94 Å), SER411 (2.80 Å), and ARG413 (2.40 Å), indicating a role in extrinsic apoptotic pathway regulation. Figure 11 shows that AFP interacts with isorhamnetin 3-*O*-rutinoside-7-glucoside and forms two H-bonds, LEU365 (2.35 Å) and VAL367 (2.45 Å). Figure 12 highlights isorhamnetin triglucoside in complex with TNF-α, anchored by interactions with GLN125 (2.68 Å), suggesting inhibition of inflammatory signaling. The box plots accompanying each figure visually reinforce the binding affinity trends, with lower binding energies indicating stronger and more stable interactions. These figures collectively support the therapeutic potential of glycosylated flavonoids as multi-target agents acting on apoptotic and inflammatory pathways.

**Table 7:** Docking scores (binding energies in kcal/mol) of the compounds with apoptosis and cancer-related targets.

Compound	BCl-2	BAX	Caspase-3	Caspase-8	AFP	TNF-α
Citric acid	− 5.1	− 5.3	− 4.6	− 4.7	− 5.9	− 5.2
Malic acid	− 4.4	− 4.6	− 4.8	− 4.9	− 5.1	− 4.3
Galloylglucose	− 7.0	− 7.1	− 6.5	− 7.1	− 7.1	− 6.7
Gentisic acid glucoside	− 6.6	− 6.8	− 6.4	− 6.7	− 7.6	− 7.8
Gentisic acid glucuronide	− 6.7	− 8.0	− 6.5	− 6.4	− 7.9	− 7.2
Galloyl−pentosylglucose	− 8.5	− 7.3	− 7.9	− 8.3	− 8.4	− 8.7
Gentisic acid pentoside	− 6.5	− 7.0	− 6.0	− 6.2	− 7.2	− 7.6
Galloylpentose	− 7.1	− 7.5	− 7.2	− 7.6	− 7.7	− 7.4
Dihydrocaffeic acid sulphate	− 6.0	− 6.0	− 5.6	− 6.2	− 7.0	− 6.6
Galloylquinic acid	− 7.2	− 7.0	− 6.2	− 6.6	− 7.3	− 6.8
Gentisic acid dipentoside	− 8.9	− 7.9	− 8.0	− 7.5	− 8.7	− 8.9
Gentisic acid	− 5.7	− 5.7	− 4.7	− 5.1	− 6.9	− 5.6
Ferulic acid 4-*O*-glucuronide	− 5.6	− 6.4	− 5.0	− 5.8	− 6.0	− 6.6
Isorhamnetin 3-*O*-rutinoside− 7-glucoside	− 10.4	− 8.1	− 9.0	− 8.5	**− 10.1**	− 9.6
Isorhamnetin malonyl- diglucoside	− 10.3	− 8.4	− 9.3	− 9.1	− 9.4	− 10.5
Quercetin-*O*-pentoside-*O*-deoxyhexoside-hexoside	**− 10.5**	**− 10.2**	− 9.4	**− 10.5**	− 9.9	− 10.4
Isorhamnetin-*O*-deoxyhexosyl-pentosyl-hexoside	− 10.2	− 8.4	**− 9.7**	− 9.6	− 9.9	**− 10.6**
Isorhamnetin 3-*O*-rutinoside	− 8.7	− 7.7	− 7.7	− 9.2	− 8.6	− 8.9
Isorhamnetin 3-*O*-glucoside	− 9.0	− 9.0	− 8.3	− 9.0	− 8.4	− 9.2
Dicaffeoyl quinic acid	− 9.0	− 8.1	− 7.1	− 8.4	− 7.8	− 8.4
Apigenin 7-*O*-glucoside	− 7.6	− 7.9	− 7.1	− 7.3	− 8.3	− 8.2
Sorafenib (Control)	− 8.1	− 7.8	− 7.8	− 8.0	− 9.8	− 9.5

## Discussion

It is worth mentioning to refer that out of the 27 metabolites characterized in this report by LC/MS, 9 metabolites only were identified before from *H. scoparium*, while the remaining 18 ones were for the first time from the *Haloxylon* genus. The metabolites previously reported are gentisic acid^61^, isorhamnetin triglycoside (rhamnosyl-pentosylglucoside)^55^, apigenin 7-*O*-glucoside^62^, isorhamnetin-*O*-hexoside-*O*-deoxyhexosyl-hexoside^52^, isorhamnetin-*O*-hydroxyferuloylhexoside-*O*-malonylhexoside^52^, quercetin-*O*-pentoside-*O*-deoxyhexoside-hexoside^52^, isorhamnetin-*O*-deoxyhexosyl-pentosyl-hexoside^52^, isorhamnetin 3-*O*-rutinoside^52^, and isorhamnetin 3-*O*-glucoside^52^.

While there are few direct reports on the anticancer activity of *H. scoparium*, indirect evidence from other close relatives of the *Haloxylon* genus and the broader Amaranthaceae/Chenopodiaceae family suggests its potential as a therapeutic compound. Methanol extract of the *H. salicornicum* was reported to induce apoptosis in cancer cells with DNA damage in the morphology of the cells. This plant possesses similar phytochemical profiles to *H. scoparium*^63^. Ethnopharmacological history also documents the traditional use of *H. scoparium* in the treatment of inflammatory disease^9,64^, suggesting probable immunomodulatory activity that could be implicated in antitumor action. To achieve our goal, firstly we detected its cytotoxic effect on the HepG2 cell line. The results showed that the calculated IC_50_ was found to be 282.4 ± 35.88 µg/ml, which did not meet the NCI’s stringent cut off for the *in vitro* activity (IC_50_ ≤ 30 µg/mL)^63,65^. The fact that it exhibited significant antitumor activity *in vivo* suggests that its mechanism of action may be beyond direct cytotoxicity. This disparity highlights a well-reported limitation of *in vitro* models, which fail to reflect the complex tumor microenvironment, metabolic activation, and systemic immune response that are all components of a compound’s overall therapeutic potential *in vivo*^66^. The majority of natural products possess multi-target mechanisms of anticancer activity, e.g. immunomodulation, anti-angiogenesis, or inhibition of tumor-stroma interactions—that are impossible to capture in monolayer cell cultures^67,68^. Thus, while *in vitro* screening is giving useful initial information, *in vivo* efficacy of the extract supports its promise for consideration in future mechanistic and preclinical studies. Based on these findings and the literature, it is determined that the extract is non-toxic *in vivo* at a dose of 200 mg/kg b.wt., orally. Herein, the HCC model was established using DEN, which is considered a liver toxin that causes liver cancer^69^. Development of HCC followed by treatment with *H. scoparium* extract showed a substantial reduction in body weight loss compared to the DEN group. Correspondingly, at the end of the experiment, 83.33% and 91.67% of mice survived in DEN and DEN/*H. scoparium* groups, and no death was detected in the normal or *H. scoparium* group. After sacrificing, the liver weight in the DEN group was less than the post-treated group (*p* = 0.045). DEN exposure resulted in the release of stress-specific enzymes in the blood, as shown by substantial increases in liver functional markers such as ALT, AST, bilirubin, and ALP^21,70^. In this study, the DEN group revealed an increase in the AST, ALT, ALP, and TBILR levels compared to other groups. This is due to severe hepatocyte necrosis, bile duct proliferation, and cholestasis, leading to both hepatocellular damage and impaired bile excretion. However, the TBILR, ALP, and AST were decreased in DEN/*H. scoparium* group relative to the DEN group, indicating liver recovery. The release of cytochrome-C or other caspase-activating factors from the mitochondrial intermembrane space into the cytoplasm is a cornerstone of the intrinsic apoptosis pathway. This release is dependent upon the mitochondrial permeability transfer pore. The apoptosome is then produced in the cytoplasm that activates caspase-9, and then activates the effector caspase cascade^71,72^. The level of Bcl-2 as a carcinogenic marker was significantly increased in the DEN group compared to other groups. In addition, apoptotic markers BAX, Caspase-3, and Cas-8 were significantly increased in DEN/*H. scoparium*-treated group compared to the normal and DEN groups. AFP protein is reactivated in a majority of hepatocellular carcinoma (HCC), its expression level was inversely related to the induction of the apoptotic pathway that was chosen as a target of investigation because it is a key mechanism regulating hepatocellular carcinoma growth, is commonly targeted by plant-derived anticancer agents, and comprises discrete, measurable molecular markers (BAX, BCl-2, caspases) that are measurable in stored tissue. Abnormal regulation of apoptosis is involved in tumor survival and resistance, and reconstitution of apoptotic pathways is a proven therapeutic target^73,74^. In the current study, the DEN group showed a remarked increase in AFP expression compared to other groups. In the post-treated group, there was a noteworthy decrease in AFP expression. TNF-α could be considered an endogenous cancer promoter because it induces cancer cell development, proliferation, invasion, metastasis, and tumor angiogenesis^75^. Nevertheless, it is considered a key inflammatory mediator that triggers immune responses^70^. The serum TNF-α was significantly increased in the DEN group (322.44 ± 11.2) compared to the normal group (0.07 ± 0.02). Besides, the TNF-α in the post-treated group was significantly decreased (73.98 ± 2.7) compared to the DEN group. Histopathological examination of the normal group showed a preserved hepatic lobular architecture with the absence of degeneration, inflammation, or fibrosis. On the other hand, the *H. scoparium* group had a preserved hepatic lobular architecture with focal cholestasis and mild hepatocytic hydropic changes. In the DEN group, there was HCC with a focal acinar formation and bizarre-shaped hyperchromatic nuclei with focal cholestasis and distorted lobular pattern. However, the post-treated group demonstrated focal dysplasia, few apoptotic figures, and cholestasis. IHC results showed a substantial elevation of Caspase-3 expression in the DEN/*H. scoparium* group compared to the normal group, which was consistent with qPCR. However, the immunohistochemical analysis of Caspase-3 in the DEN group showed a remarkable increase in the protein expression level that was higher than the treated group (DEN/*H. scoparium*). This result may be attributed to the direct involvement of Caspase-3 in promoting tumor repopulation or a non-apoptotic role for caspases that includes caspase-mediated stimulation of differentiation, dedifferentiation, and T-cell activation^76^. The constitutive phytoconstituents, which were identified in the methanolic extract of *H. scoparium* by LC-MS/MS, displayed significant anticancer properties according to previous research reports. Among all phenolic acid derivatives, ferulic acid-based derivatives had a notable ability in cancer cell proliferation inhibition along with apoptosis induction and oxidative stress manipulation. Laboratory studies displayed its potential cancer-fighting capabilities by controlling tumor advancement through PI3K/Akt and MAPK signaling pathways to become a possible treatment against different cancers^77^. Similarly, the chlorogenic acid derivative, dicaffeoyl quinic acid, exhibited cytotoxic effects against different cancer cells by inducing cell cycle arrest and apoptosis^78^. On the other hand, quercetin and isorhamnetin derivatives were proved to be two significant flavonoidal candidates in the extract. The pro-apoptotic, anti-metastatic, and anti-inflammatory effects of quercetin triglycoside, as a key compound, was explained to promoteapoptosis signaling pathways through modulation of NF-κB and p53, together with caspase activation^79^. Previous reports clarified how are the different isorhamnetin-based glycosides such as tetraglycoside, triglycoside, and rutinoside, exhibit cancer cell antiproliferative and antimigrative properties specifically in lung, liver, and breast cancer cells through their ability to control oncogenic signaling directions, which involve both STAT3 and Wnt/β-catenin^80,81^.

## Conclusions

Current findings showed that *H. scoparium* leaf extract protected the liver and fought cancer cells during hepatocellular carcinoma development with DEN exposure. The DEN-induced liver damage was confirmed by elevation of the ALT, AST, ALP, TBILR, AFP, and TNF-α levels and liver tissue changes such as hepatocyte necrosis, together with bile duct proliferation and distorted hepatic structure. The use of *H. scoparium* extract following treatment resulted in both enhanced liver functional parameters and decreased AFP and TNF-α concentrations, while maintaining normal hepatic tissue structure. The anticancer effects of *H. scoparium* extract were associated with enhanced apoptotic activities revealed through elevations in BAX, while Caspase-3 and Caspase-8 expression increased too.

The results validate *H. scoparium* extract’s potential function as a preventive and therapeutic solution for HCC. The identification of various compounds in *H. scoparium* recommended its potential as a promising biosafe natural source of anticancer agents. Future studies should focus on isolation such bioactive compounds, elucidating their individual specific mechanisms of action, and exploring their potential for therapeutic applications in cancer treatment.

*In silico* molecular docking analysis supported the *in vitro* and *in vivo* findings, revealing strong binding affinities for the major glycosylflavonoids, such as quercetin and isorhamnetin glycosides, toward cancer-related proteins, including BCL-2, BAX, Caspase-3, Caspase-8, AFP, and TNF-α. These interactions were stabilized by multiple hydrogen bonds and π–π interactions, especially at key residues involved in apoptotic and oncogenic signaling pathways. Such evidence underscores the multi-target potential of *H. scoparium* phytoconstituents in modulating cancer progression mechanisms.

## Limitations and prospects

The hepatoprotective, along with anticancer effects found in this research, need additional consideration for addressing existing limitations. Future investigations should follow pathways of apoptosis together with oxidative stress, inflammation, and angiogenesis to determine the full therapeutic capacity of *H. scoparium*. Long-term chronic exposure research protocols will be useful to determine what sustained effects arise from *H. scoparium* exposure. Future prospective studies should be performed to extend research with *H. scoparium* in liver disease model tests over time to evaluate its ability to stop HCC development. Therapeutic effectiveness and biosafety need additional research regarding *H. scoparium*’s proper dosage levels along with pharmacokinetic testing of its distribution and metabolism patterns, and toxicity evaluation before its clinical use would be approved.

## Data Availability

All data generated or analyzed during this study are included in this published article and its supplementary information files.

## Abbreviations

HCC: Hepatocellular carcinoma
LC/ESI-MS/MS: Liquid chromatography/electrospray ionization-mass spectrometry/ mass spectrometry
HepG2: Hepatocellular carcinoma
DEN: Diethylnitrosamine
ALT: Alanine aminotransferase
AST: Aspartate aminotransferase
ALP: Alkaline phosphatase
TBILR: Total direct bilirubin
TNF-α: Tumor necrosis factor alpha
LC-UV-MS/MS: Liquid chromatography-ultraviolet-mass spectrometry
BCL-2: B-cell lymphoma-2
AFP: Alpha-fetoprotein
i.p.: Intraperitoneally
PCR: Polymerase chain reaction
RNA: Ribonucleic acid
cDNA: Complementary deoxyribonucleic acid
qPCR: Quantitative polymerase chain reaction
TBST: Tris-buffered saline with Tween^®^ 20
PBS: Phosphate-buffered saline
SEM: Standard error of the mean
ANOVA: Analysis of variance
R_t_: Retention time
IC_50_: Half maximal inhibitory concentration
